# Suicidal ideation, attempts and help-seeking behaviors among adolescent girls in, Southwest Ethiopia: a community-based cross-sectional study

**DOI:** 10.1186/s13034-025-00996-0

**Published:** 2025-11-27

**Authors:** Tesfaye Segon, Aman Dule, Dagmawit Alemayehu, Mekidem Aderaw, Mamaru Melkam, Techilo Tinsae, Girum Nakie, Getasew kibralew, Gebresilassie Tadesse, Tirusew Wondie, Endris Seid, Mulat Kassa, Gebeyaw Molla Kassie, Zelalem Belayneh, Yigreme Ali, Alemayehu Molla

**Affiliations:** 1 Department of Psychiatry, College of Medicine and Health Science, Injibara University, Injibara, Ethiopia; 2 Department of Psychiatry, College of Health Science, Mettu University, Mettu, Ethiopia; 3 Department of psychiatry, College of Medicine and Health Science, University of Gondar, Gondar, Ethiopia; 4 Department of Psychiatry College of Medicine and Health Science, Debre Markos University, Debre Markos, Ethiopia; 5 Department of Psychiatry, College of Medicine and Health Science, Dilla University, Dilla, Ethiopia; 6 Department of Psychiatry, College Health Science Woldia University, Woldia, Ethiopia; 7 Faculty of Medicine, and Nursing and Health Sciences, School of Primary and Allied Health care, Monash University, Melborn, Australia; 8 School of Health, Faculty of Medicine and Health, University of New England, Armidale, NSW 2351 Australia

**Keywords:** Suicidal ideation, Suicidal attempt, Suicidal plan, Gender based violence, Premenstrual dysphoric disorder, Help-Seeking, Adolescent girls, Ethiopia

## Abstract

**Background:**

Suicide poses a significant global health challenge and is a leading cause of death among adolescent girls. Although suicidal ideation and attempts are known to increases sharply during female adolescence, and help-seeking behaviors are especially rare in low-income countries, no studies have been conducted on this population in Ethiopia. This study aimed to assess the magnitude, factors related to suicidal ideation and attempts, and help-seeking behaviors.

**Methods:**

A community-based cross-sectional study was carried out in Illu Abba Bor Zone, involving 847 participants selected through multistage cluster sampling. Data, collected using interviewer-administered WHO Composite International Diagnostic Interview (CIDI) and General Help-Seeking Questionnaires, were analyzed using SPSS Version 26.0. Bivariate and multivariate analyses were performed to identify factors associated with the outcomes.

**Results:**

Of the 847 sampled girls, 810 participated, yielding a response rate of 95.6%. The prevalence of suicidal ideation, suicidal plan and attempts was 25.3%, 19.8%, and 16.1%, respectively. Their help-seeking behaviors for suicidality were 14.6%. Risk factors for suicidal ideation included experiencing gender-based violence (AOR = 3.68, 95% CI: 1.94, 6.46), anxiety (AOR = 2.04, 95% CI: 1.42, 4.21), food insecurity (AOR = 1.93, 95% CI: 1.19, 3.48), and premenstrual dysphoric disorder (AOR = 3.54, 95% CI: 1.78, 6.21). For suicidal attempts, associated factors were depression (AOR = 3.14, 95% CI: 2.01, 6.52), gender-based violence (AOR = 4.56, 95% CI: 2.12, 9.34), social phobia (AOR = 1.84, 95% CI: 1.27, 3.13), and a family history of suicide (AOR = 2.14, 95% CI: 1.41, 3.65).

**Conclusions:**

Findings indicate that one in four adolescent girls experienced suicidal ideation, one in six made attempts, but rates of help-seeking behaviors were low. Immediate intervention is crucial; it is essential to design and implement comprehensive, multi-level strategies targeting suicide prevention. Promoting community mental health, help-seeking behaviors for suicidality, and reducing violence against girls are vital steps needed to tackle mental health issues.

## Background

Adolescence is a crucial transitional phase between childhood and adulthood, marked by significant growth and change, second only to infancy [1]. Approximately 85% of adolescents globally reside in low- and middle-income countries [2]. In Sub-Saharan Africa, adolescents make up 20–30% of the population, with several countries experiencing a larger and growing adolescent demographic compared to other regions of the world [3, 4]. In Ethiopia, roughly one in four individuals is an adolescent [5].

During this stage, there is a marked change in biological, cognitive, and social experiences, especially among females, including significant increases in the occurrence of emotional and physiological responses to interpersonal stress that results in suicidal thoughts and behaviors among adolescent girls [6]. During this period, girls adopt additional gender-specific roles, and as they navigate individuation and exploration, their options may become limited, which might culminate in suicide. Notably, there is a sharp rise in suicidal thoughts and actions among teenage girls 7. Suicidal ideation is any self-reported desire to harm oneself that is not accompanied by any preparatory behavior. Suicidal planning means formulating a specific, feasible, and intentional method to carry out the action. This often involves steps taken to acquire the means or set the timing. The suicidal attempt is a nonfatal outcome that is instigated and perpetrated by the person in question and culminates in self-harm [8, 9].

A recent study reported that suicidal thoughts and behaviors rise dramatically during this developmental stage [10]. Globally, suicide accounts for approximately 6% of all adolescent deaths [11] and 70% of global violent deaths in women [12]. It is the second leading cause of death among female adolescents [13]. Nearly, 800,000 adolescents die by suicide each year, with over 79% occurring in LMICs [14]. Studies report, girls are more likely than boys to attempt suicide and report suicidal ideation [15]. Approximately 53% of American adolescents experience suicidal thoughts [16]. A study done in U.S.A, 22% of adolescent girls report suicidal ideation, 9.3% attempt suicide annually [17]. Another cross-sectional study in Turkey indicated that 13.2% of adolescent girls have attempted suicide [18]. The prevalence of suicidal attempts and ideation among adolescent girls in the community ranged from 13% to 21% [19]. However, compared to high-income countries relatively little is known about the epidemiology of adolescent suicide and suicidal behaviors in LMICs.

Adolescent suicidality is a significant mental health issue, with suicide rates increasing despite prevention efforts especially LMICs [20]. Many adolescents who experience suicidal thoughts or behaviors face barriers to accessing mental health services, and these barriers discourage help-seeking behavior; only about 28% receive professional help [21]. According to WHO data, only 10% of adolescents who attempt suicide seek professional help [22]. Research suggests that fewer than half of adolescents who died by suicide received prior mental health care [23]. A study in Taiwan found that among adolescents with a lifetime history of suicidal behavior, 5.5% sought psychiatric services and 5.0% sought help from mental health workers. For those with suicidal behavior in the past week, 17.4% sought psychiatric services, while none approached mental health workers [24]. A study conducted in America shows that 55% of individuals who commit suicide had no primary care contact, and 68% had no contact with mental health services [25]. Adolescent suicide help-seeking in Low- and Middle-Income Countries (LMICs) is critically low due to a complex interplay of systemic and cultural barriers. These include insufficient mental health resources, widespread under-diagnosis and under-treatment, and a lack of awareness regarding available psychiatric care. Culturally, help-seeking is aggressively hindered by pervasive stigma, deeply rooted cultural taboos surrounding suicide, poor mental health literacy, and the fear of negative outcomes [26, 27]. Even with accessible psychiatric services and universal health coverage, stigma and lack of information may explain low help-seeking rates [26]. Clarifying the roles of mental health care provision and help-seeking is therefore essential for suicide prevention. Despite high suicide rates in Ethiopia, many people do not seek professional help for suicide, and no evidence exists on suicidal behaviors and help-seeking among adolescent girls. This study addresses suicidal concerns and help-seeking behaviors in this population.

Adolescent mental health is significantly impacted by suicide, with emotional, physical, and financial consequences. Adolescent girls are more susceptible to anxiety, mood swings, and internalizing disorders, leading to depression and other mental health issues [28]. Survivors may experience trauma, leading to long-term negative effects on their well-being [29]. The impact extends beyond individuals, affecting friends, family, coworkers, and the community [30, 31]. Despite different studies done in Ethiopia on suicide in various populations, adolescent females have received less attention in Ethiopian studies, making understanding suicidal ideation and attempt patterns, and their help-seeking behaviors is crucial for distributing health resources, designing policies, and implementing effective treatment and preventive interventions.

Suicide is a serious and preventable public health issue, with timely and low-cost intervention [32]. Different biological, psychological, and social factors affect adolescent girls’ suicidal ideation and attempts. Among these factors are experiences of bullying [33], sexual, emotional, and physical abuse [34]. In addition, childhood physical and sexual abuse appears to be risk factors for future suicide attempts [35]. Other important risk factors includes family situation, interpersonal conflict, disturbed functioning in social roles, hopelessness, substance use, and mental disorders [36, 37].

Despite, adolescent girls are at higher risk for suicidal behavior, and low rates of help-seeking for suicide particularly in vulnerable populations such as adolescent girls in LMICs, less attention is given regarding suicidality and helpseeking behaviour among adolescent girls in Ethiopia. Therefore, this study was designed to address this gap by assessing the magnitude and risk factors of suicidal plan, ideation, attempts, and help-seeking behaviors among adolescent girls in Southwest Ethiopia. The results will have important clinical and policy implications, providing policymakers, planners, and health professionals with valuable insights to design strategies for preventing suicidal behaviors, and promoting their help-sought among adolescent girls. The findings of this study will also contribute to future research in this field.

## Methods and materials

### Study area, design and populations

A community-based cross-sectional study design was conducted among adolescent girls (10–19) from October to December 2023. The study was conducted in the Ilu Ababore zone, Oromia regional state, Southwest Ethiopia. The Zone is located 600 km southwest of the capital city of Ethiopia, Addis Ababa. It has a zone with 15 administrative woredas. Based on the 2007 Census conducted, the zone has a total of 2,271,609 men and 634,623 women in the population. This zone has 272,555 households, averaging 4.67 persons per household and comprising 263,731 housing units. The zone has one comprehensive specialized hospital and 41 health centers. The outpatient department of Mettu Karl Comprehensive Specialized Hospital offers psychiatry services, while no hospitals in the Illu Ababore Zone provide inpatient psychiatric care (unpublished Illu Ababore zone health office report 2023).

All adolescent-age girls (10–19 years) residing in Illu Ababore zone and accessible throughout the data collection periods were the source population for this study, and adolescent girls who were living for ≥ 6 months and older in the Illu Ababore zone during the data collection period were the study population for this study. All adolescent girls who were living in Illu Ababore zone were included in this study, and individuals who were unable to communicate due to acute illness during data collection time and those who had hearing and speech impairment were excluded from this study.

### Sample size determination and procedures

#### Sample size determination

The sample size was determined using a single population proportion formula with a 95% confidence interval, a 5% marginal error, and an estimated proportion (P) of 50% since no previous studies were conducted on suicidal ideation and attempts among adolescent girls in Ethiopia. Therefore the sample size was 385.

#### Sampling procedure

The study used a multistage cluster sampling approach to select participants from fifteen woredas in the Illu Abba Bor Zone. The design effect was set at 2 due to limited funding and resources, and a non-response rate of 10% was added. Participants were selected through a lottery method, reaching out to three woredas (woreda is an administrative division or district in Ethiopia, which is further subdivided into kebeles),15 kebeles (kebele is a term used in Ethiopia for the smallest administrative unit or neighborhood within a woreda) in each cluster, and on average 2230 households in a kebele. All girls aged 10–19 were included. The final sample size was allocated proportionally among selected kebeles based on household numbers, with one household selected if multiple households were found in a house.

#### Data collection instruments

Data were collected using a structured face-to-face interviewer-administered questionnaire. An outcome variable, suicide ideation, and attempt was assessed by using the World Health Organization Composite International Diagnostic Interview (CIDI), which was a standard and cross-culturally validated tool [38].

The General Help-Seeking Questionnaire (GHSQ) assessed help-seeking behaviors for suicidal problems. Specifically, it measured the likelihood of participants seeking help from various sources (mental health professionals, psychiatrists, non-psychiatric medical clinics, social workers, psychologists, psychotherapists, general practitioners, family, friends, the internet, school campuses, workplaces, religious and traditional healers, and nobody) in response to hypothetical suicidal ideation and attempts [39]. The Cronbach’s alpha for actual help sought for suicidal problems was 0.83 [40].

Explanatory variables were measured using standardized and validated tools, including the Patient Health Questionnaire 9 modified for adolescents (PHQ-9 A), which was used to measure depression. The tool consists of 9 items, including the following response options: 0 “not at all,” 1 “several days,” 2 “more than half the days,” and 3 “nearly every day”. The sum of items could range between 0 and 27, and having a score of greater than or equal to 10 is considered to have depression [41]. In this study, Cronbach’s alpha was 0.79.

The ASIST-GBV (Assessment Screen to Identify Survivors Toolkit questionnaire for Gender Based Violence) questionnaire, validated in Ethiopia, was used to assess gender-based violence (GBV) in humanitarian settings. It consists of questions about emotional, physical, and sexual (forced sex, coercive sex for survival, forced pregnancy, and forced marriage) violence. It has high internal consistency with a Cronbach’s alpha 0.77 [43]. The presence of GBV is indicated by participants who provide at least one ‘yes’ response [43]. Anxiety was assessed using the Generalized Anxiety Disorder 7 (GAD-7). The scale uses a four-point Likert scoring system, where responses are scored as “not at all” (0), “several days” (1), and " more than half the days " (2), and " nearly every day " (3). A score of greater than or equal to 10 is considered to have anxiety [42].

Social phobia was assessed by using Social Phobia Inventory (SPIN). It was validated in Nigeria, and used in Ethiopia. A score of 20 and above on SPIN considered as having social phobia [43]. Food insecurity was measured using the Household Food Insecurity Scale (HFIAS), which determined how frequently respondents went hungry due to a lack of food in the home over the previous 30 days. Frequency ratings ranged from 0 to 3: with 0 indicating “nonoccurrence,” 1 indicating “rarely” once or twice in the previous four weeks, 2 indicating “sometimes” three to ten times in the last four weeks, and 3 indicating “frequently” more than ten times in the last month [44]. In this study, scores of 0 were classed as no and labeled as 0, while scores of 1–9 were classified as yes and labeled as yes, and food insecure [45].

Premenstrual dysphoric disorder (PMDD) was assessed by using DSM-5. Adolescent girls have PMDD if they reported at least five DSM-5 diagnostic criteria symptoms in most of their menstrual cycles. The symptoms had to be present in the final week before the start of menstruation [46].

ASSIST (Alcohol, Smoking, and Substance Involvement Screening Test) questionnaire adapted from the WHO was used to assess substance use [47]. The Oslo 3 Social Support Scale measures the perceived level of social support. The scale divides the level of social support into three categories: poor (3–8), moderate (3–14), and strong (12–14) [48]. Socio-demographic factors and other clinically related factors including family history of suicide, comorbid medical illness, and family history of mental illness were collected by using structured questionnaires.

### Data collection procedure and data quality control

The study used standardized assessment tools and a pre-test conducted in Bedele town with 5% (*n* = 43) of the total sample. The questionnaire was translated into Amharic and Afan Oromo, and back-translated by experts. Data collectors and supervisors received training on the tools and ethical considerations. Close supervision was maintained throughout the data collection period, with investigators reviewing completed questionnaires for consistency. Data was gathered through face-to-face interviews using pre-tested, structured, and standardized questionnaires.

### Data processing and analysis

The data was entered into Epi-Data version 4.6.02 and analyzed with SPSS version 26.0 software. The data distribution was summarized using descriptive statistics, which included frequency and percentage. The data was given in tables. Bivariate and multivariable logistic regression models were used. Variables having a p-value of less than 0.2 in the bivariate analysis were included in the multivariate regression analysis to account for any confounding effects. The Hosmer-Lemeshow test was used to assess the model’s goodness of fit. In the multivariable logistic regression analysis, statistically significant related factors were identified using Adjusted Odds Ratios (AOR) with 95% confidence intervals (CI) and a p-value < 0.05.

Ethical approval for this study was obtained from the Ethical Review Committee (ERC) of Mettu University Department of Psychiatry. Informed written consent was obtained from each participant after providing a clear explanation of the study’s purpose and objectives. Participants were assured that their participation was voluntary and that they had the right to withdraw at any time without any negative consequences. Written informed consent was sought and obtained from their parents or guardian for adolescent girls under 18 years old. To ensure confidentiality, participants’ identities were kept anonymous, and personal information was treated with strict confidentiality throughout the study.

## Results

### Socio-demographic characteristics of the participants

A total of 810 participants were involved voluntarily in this study, resulting in an overall response rate of 95.6%. The age of the respondents ranged from 10 to 19 years with a mean age of 15.1 (standard deviation ± 2.83). Among the study participants, 300 (37.0%) were Orthodox religious followers, 664 (81.9%) were living with both parents, and 88 (10.9%) were living with one parent. The majority of the participants 433 (53.5%) were primary school students, 702 (86.7%) of both parents were alive, and 37 (4.5%) of both parents were not alive (Table 1).

**Table 1:** Distribution of socio-demographic characteristics among adolescent girls in, Southwest Ethiopia, 2023(*n* = 810)

Variables	Categories	Frequency	Percent (%)
Age	10–14	367	45.3
	15–19	443	54.7
Religion	Orthodox	300	37.0
	Muslim	264	32.6
	Protestant	201	24.8
	Others	45	5.6
Living arrangements	With both parents	664	81.9
	With one parent	88	10.9
	Alone	21	2.6
	Relatives	37	4.6
Educational status	No formal education	38	4.7
	Primary education	433	53.5
	High school and above	339	41.8
Father’s educational status	Unable to read and write	74	9.1
	Able to read and write	68	8.4
	Primary	372	46.0
	Secondary and above	296	36.5
Mothers educational status	Unable to read and write	97	12.0
	Able to read and write	86	10.6
	Primary	359	44.3
	Secondary and above	268	33.1
Parents living status	Both alive	702	86.7
	Both not alive	37	4.5
	Only one parent alive	71	8.8

*Catholic, Waqefeta, and Adventist

### Clinical characteristics of the participants

Of the study participants, 463 (57.2%) of them experienced gender-based violence, and 327 (40.4%) were food insecure. Among study participants, 321 (39.6%) had social phobia, 61 (7.5%) had a family history of suicide, and 291 (35.9%) of adolescent girls had PMDD (Table 2).

**Table 2:** Distribution of clinical related factors among adolescent girls in, Southwest Ethiopia, 2023(*n* = 810)

Variables	Categories	Frequency	Percent (%)
Gender based violence	Yes	463	57.2
	No	347	42.8
Family history of mental illness	Yes	101	12.5
	No	709	87.5
Food insecurity	Yes	327	40.4
	No	483	59.6
Comorbid medical illness	Yes	56	6.9
	No	754	93.1
Family history of suicide	Yes	61	7.5
	No	749	92.5
Social phobia	Yes	321	39.6
	No	489	60.4
PMDD(Premenstrual dysphoric disorder)	Yes	291	35.9
	No	529	64.1

### Psychosocial and behavioral characteristics of study participants

Among study participants, 391 (48.3%) of them had depression, 423 (52.2%) had anxiety, and 42 (5.2%) were current alcohol drinkers. Regarding social support, 320 (39.5%) had moderate social support, and 192 (23.7%) of them had poor social support (Table 3).

**Table 3:** Characteristics of psychosocial and behavioral factors among adolescent girls in, Southwest Ethiopia, 2023(*n* = 810)

Variables	Categories	Frequency	Percent (%)
Depression	Yes	391	48.3
	No	419	51.7
Anxiety	Yes	423	52.2
	No	387	47.8
Social support	Strong	298	36.8
	Moderate	320	39.5
	Poor	192	23.7
Ever use of khat	Yes	17	2.1
	No	793	97.9
Current use of khat	Yes	11	1.4
	No	799	98.6
Ever use of alcohol	Yes	51	6.3
	No	759	93.7
Current use of alcohol	Yes	42	5.2
	No	768	94.8

### Prevalence of suicidal ideation and associated factors among adolescent girls

In the current study, the overall prevalence of suicidal ideation among adolescent girls for the last 12 months was 25.3% (95% CI: 23.5%−28.4%). Variables with a P-value < 0.2 in binary logistic regression analysis were included as candidates for multivariable logistic regression analysis. In the multivariable binary logistic regression model GBV, having anxiety, food insecurity, and PMDD (premenstrual dysphoric disorder) was found to be significantly associated with suicidal ideation with 95% CI with a P-value less than or equal to 0.05 among adolescent girls in Ethiopia.

The odds of suicidal ideation were 3.7 times more common among adolescent girls who had experienced gender-based violence as compared to those who had not experienced gender-based violence (AOR = 3.68, 95% CI: 1.94, 6.46). Participants with anxiety symptoms were twice as likely to have suicidal ideation as adolescent girls who did not (AOR = 2.04, 95% CI: 1.42, 4.21). Participants who were food insecure were 1.93 times more likely to have suicidal ideation as compared to their counterparts (AOR = 1.93, 95% CI: 1.19, 3.48). Moreover, those who had PMDD were about 2.54 times more likely to develop suicidal ideation when compared with those who had no PMDD (AOR = 3.54, 95% CI: 1.78, 6.21) (Table 4).

**Table 4:** Bi-variables and multi-variables regression analysis between suicidal ideation and explanatory variables among adolescent girls in, Southwest Ethiopia, 2023 (*n* = 810)

Variables	Category	Suicidal ideation		COR(95% CI)	AOR(95% CI)
		Yes	No		
Age	10–14	88	279	1	1
	15–19	117	326	1.14(0.68–1.9)	0.77(0.49–1.18)
Living arrangements	Alone	8	13	2.02(1.01–3.53)	1.34(0.72–2.75)
	Single parents	30	58	1.69(1.00–2.56.00.56)	1.12(0.54–1.74)
	Relatives	12	25	1.57(0.47–2.01)	0.66(0.30–1.42)
	Both parents	155	509	1	1
Depression	Yes	140	251	3.04(1.62–5.59)	1.74(0.83–3.51)
	No	53	354	1	1
Social support	Poor	80	112	3.02(1.34–5.21)	1.35(0.79–2.05)
	Moderate	68	252	1.14(0.67–2.01)	0.73(0.46–1.15)
	Strong	57	241	1	1
GBV	Yes	167	296	4.59(2.41–8.36)	3.68(1.94–6.46)^**^
	No	38	309	1	1
Anxiety	Yes	151	272	3.42(1.79–4.21)	2.04(1.42, 4.21)^*^
	No	54	333	1	1
Comorbid medical illness	Yes	20	36	1.71(0.94–2.13)	1.21(0.58–2.32)
	No	185	569	1	1
Social phobia	Yes	116	205	2.54(1.73–3.29)	1.46(0.80–2.83)
	No	89	400	1	1
Family history of suicide	Yes	22	39	1.74(0.98–2.16)	1.17(0.63–1.95)
	No	183	566	1	1
Food insecurity	Yes	122	205	2.87(1.58–5.47)	1.93(1.19–3.48)^*^
	No	83	400	1	1
Family history of mental illness	Yes	36	62	2.06(1.33–4.10)	1.41(0.72–2.63)
	No	166	543	1	1
PMDD	Yes	140	151	6.62(3.67–10.10)	3.54(1.78–6.21)^**^
	No	65	464	1	1

*p*-value = **<0.01; *<0.05

Hosmer-Lemeshow test = 0.74

### Prevalence and associated factors of suicidal attempts among adolescent girls

In this study, the overall prevalence of suicidal attempts among adolescent girls for the last 12 months was 16.1% (95% CI: 13.5%−19.2%). Of the study participants who had 12-month suicide attempts, 56.1% (73) of adolescent girls had a history of trying to kill themselves one time, 30.8% (40) of respondents had a history of a suicide attempt twice, and 13.1% (17) of the participants were trying to kill themselves more than two times. Of those who attempted suicide, 67.7% (89) of participants used poisoning methods to kill themselves, 20.8% (27) by hanging, and 10.0% (13) by using sharp materials. Regarding the seriousness of the suicidal attempts, 64.3% (83) of study participants reported a serious attempt to kill themselves, and it was only luck that they did not succeed, and 22.2% (29) had attempted suicide to cry for help, and 13.5% (18) did not intend to kill themselves(Table 5).

**Table 5:** Frequency distribution of suicidal ideation, suicidal plan and attempts among adolescent girls in, Southwest Ethiopia, 2023(*n* = 810)

Variables	Categories	Frequency	Percent (%)
Suicidal ideation in the last 12 month	Yes	205	25.3%
	No	605	74.7
Last 12 month suicide plan	Yes	161	19.8
	No	649	71.2
Suicidal attempts in the last 12 month	Yes	130	16.1%
	No	680	83.9
12-month frequency of suicide attempts	Once	73	56.1%
	Twice	40	30.8%
	More than twice	17	13.1%
Methods used for suicide attempts	Hanging	27	20.8%
	Poisoning	88	67.7%
	Using sharp material	13	10.0%
	Others	2	1.5%
Which describes your suicide?	A serious attempt	83	64.3%
	Tried to kill me	29	22.2%
	I did not intend to die	18	13.5%

In a multivariable logistic regression analysis, having depressive symptoms, GBV, social phobia, and a family history of suicide was found to be significantly associated with suicidal attempts with a 95% CI at a P-value less than or equal to 0.05.

The odds of suicidal attempts were three times higher among adolescent girls who had depressive symptoms than among participants who had no depressive symptoms (AOR = 3.14, 95% CI: 2.01, 6.52). Adolescent girls who had experienced gender-based violence were about 4.6 times more likely to have suicidal attempts than participants who had not experienced gender-based violence (AOR = 4.56, 95% CI: 2.12, 9.34). Those participants who had social phobia were 1.8 times more likely to have suicidal attempts than participants who had no social phobia (AOR = 1.84, 95% CI: 1.27, 3.13). Furthermore, the odds of having suicidal attempts among participants who had a family history of suicide were two times higher as compared with the referent groups (AOR = 2.14, 95% CI: 1.41, 3.65) (Table 6).

**Table 6:** Bi-variables and multi-variables regression analysis between suicidal attempts and explanatory variables among adolescent girls in, Southwest Ethiopia, 2023 (*n* = 810)

Variables	Category	Suicidal attempt		COR(95% CI)	AOR(95% CI)
		Yes	No		
Age	10–14	72	295	1	1
	15–19	58	385	1.62(0.84–2.78)	0.68(0.34–1.59)
Parents living status	Both alive	104	598	1	1
	Both not alive	9	28	1.85(1.28–2.60)	1.14(0.71–2.01)
	Only one parent alive	17	54	1.81(0.98–2.84)	0.83(0.36–1.97)
Depression	Yes	104	287	5.48(3.10.93)	3.14(2.01–6.52)^**^
	No	26	393	1	1
GBV	Yes	112	351	5.83(3.58–9.20)	4.56(2.12–9.34)^**^
	No	18	329	1	1
Anxiety	Yes	94	329	2.79(1.58–4.34)	1.45(0.84–2.81)
	No	36	351	1	1
Social phobia	Yes	77	244	2.09(1.45–3.17)	1.84(1.27–3.13)^*^
	No	55	364	1	1
Comorbid medical illness	Yes	15	41	2.03(1.35–3.39)	1.53(0.68–3.21)
	No	115	639	1	1
Family history of suicide	Yes	20	41	2.83(1.71–4.53)	2.14(1.41–3.65)^*^
	No	110	639	1	1
Food insecurity	Yes	68	259	1.78(1.34–2.97)	1.52(0.57–3.10)
	No	62	421	1	1
Family history of mental illness	Yes	25	76	1.89(1.25–3.28)	1.64(0.76–3.12)
	No	105	604	1	1
PMDD	Yes	64	227	1.86(0.85–3.88)	1.54(0.59–3.25)
	No	66	436	1	1

*p*-value = **<0.01; *<0.05

Hosmer-Lemeshow test = 0.74

### Help‑seeking behaviors of suicide among adolescent girls

In the past 12 month, 14.6% of adolescent girls had help-seeking behaviors for suicidality, primarily from parents (27.5%) and intimate partners (19.4%). Additionally, spiritual sources (11.6%) and healthcare providers (9.3%) were consulted, but only 1.8% of adolescent girls sought mental health or psychiatric services.

## Discussion

Generally, higher estimates of suicidal ideation, planning, and attempts have been reported among adolescent girls [28], alongside low rates of help seeking behaviors for suicidality particularly in LMICs. So, in the current study, the magnitude of suicide ideation and attempts and their possible association with various factors, and their help-seeking behaviors were assessed among adolescent girls in Ethiopia for the first time. The findings of this study showed that the prevalence of past 12-month suicidal ideation among adolescent girls was 25.3% (95% CI: 23.5%−28.4%). The current study is consistent with studies done in Peru (26.3%) [49], and Liberia (27.0%) [50]. However, this finding is higher than previous studies done in the U.S. (22%) [17], and Myanmar (18.5%) [42]. Possible reasons for these differences between countries include differences in socioeconomic status, sociocultural conditions, and differences in availability of mental health facilities and health professionals, leading to differences in early detection and treatment [51]. For example, in Ethiopia, adolescent girls’ life opportunities are significantly limited by gender inequalities, cultural norms, and expectations of early marriage, domestic duties, and child-rearing, all of which contribute to suicidal thoughts. In contrast, adolescent girls in the U.S. have more legal rights and educational opportunities, so they are less stressed about fundamental survival and freedom, focusing more on performance and social dynamics, which can be protective against suicidal ideation [52].

The prevalence of suicidal attempts among adolescent girls in this study was 16.1% (95% CI: 13.5%−19.2%), which is in line with previous studies done in the Americas (18.6%) [53], and Samoa (17.4%) [54]. However, the proportion of suicidal attempts in this study was higher than in previous studies done in the U.S. (9.3%) [55], and Turkey (13.2%) [56]. The possible explanation for this discrepancy might be due to sociocultural factors, level of mental health awareness, attitudes towards mental illness, and study settings. For example, in Ethiopia, mental health issues are heavily stigmatized and are often viewed as a spiritual weakness or curse. This makes girls suffer in silence, as speaking out would bring shame to the family, leading to hopelessness and suicidal attempts as the only perceived escape. In contrast, the U.S. and Turkey have a much greater societal awareness and open discussion regarding to mental health [55].The other possible reasons accounting for the variations in study findings could be the differences in the measurement of suicidal behaviors as well as differences in time. For example, the above studies used the Global School-based Health Survey (GSHS), while the current study used CIDI to assess suicidal ideation and suicide attempts. On the other hand, the prevalence of suicidal attempts was lower than what was reported in Guatemala (20.2%) [57], Liberia (33.4%) [50], and Mongolia (31.3%) [58]. The discrepancy may stem from the sensitive nature of suicide; participants from various sociocultural backgrounds may be reluctant to discuss topics deemed socially unacceptable, influencing the results. Additionally, sociocultural stigma and taboos might deter individuals from reporting suicidal behaviors, thereby affecting the prevalence data [52].

In the current study, we discovered that participants who had experienced gender-based violence were 3.7 times more likely to have suicidal thoughts than those who had not. The findings are consistent with other research conducted in USA [59], Cambodia [60], Ghana [61], and Tanzania [62]. This could be because GBV has a number of implications for adolescent females, including social discrimination and stigmatization, physical handicap, school and employment absenteeism, and adolescent girls’ declining economic reliance, which leads to suicide [63]. GBV experiences and the fear of such abuse might impede gender equity for adolescent women by devaluing them and limiting their education, employment, and mobility owing to safety concerns, potentially leading to suicide [64]. Furthermore, experiencing GBV throughout adolescence has been connected to negative health and social outcomes, such as mental health problems, school dropout, substance abuse, hazardous sexual behaviors, and injury, with long-term consequences for future health and well-being, including suicide [61, 63]. GBV can lead young women on a path toward future violence and sexual risk behavior [62]. These findings indicate that designing an intervention strategy, stronger community engagement and develop treatment modalities to diminish the effects and, consequences of GBV among adolescent girls are crucial.

The current study also showed that participants who had anxiety symptoms were two times more likely to have suicidal ideation than adolescent girls who had no anxiety symptoms. This is similar to studies conducted in Taiwanese and Vietnamese [65, 66]. The possible explanation is that adolescent girls with anxiety symptoms face excessive worry that causes apprehensive expectations, restlessness, muscle tension, irritability, difficulty in falling asleep, and stress that results in suicidal behavior [65]. Moreover, anxiety symptoms become overwhelming, or experiencing distress in adolescent girls may lead to suicide as a coping strategy for emotional problems/overwhelming situations [66]. This result indicates an approach to anxiety to minimize suicidal ideation is necessary.

This study also revealed that adolescent girls who were food insecure were 1.73 times more likely to have suicidal ideation as compared to their counterparts. This is consistent with evidence from previous studies done in Ghana and Liberia [50, 61]. This could be Adolescent girls who go hungry may face heightened distraction, irritability, and increased emotional sensitivity, potentially leading to suicidal tendencies [61].

Adolescent girls with PMDD are approximately 3.54 times more likely to experience suicidal ideation than those without PMDD, as supported by studies from the USA, South Korea, and Germany [67–69]. These girls report a range of cognitive, psychological, and somatic symptoms, including heightened stress, depression, anxiety, mood swings, irritability, abdominal bloating, social withdrawal, and poor concentration, all of which contribute to an increased risk of suicidal thoughts and behaviors [67]. Consequently, adolescent girls with PMDD should be regarded as a high-risk group for suicidality, representing a significant risk factor for both the onset and exacerbation of suicidal behavior each month [69]. Therefore, timely identification and treatment of their symptoms are crucial for reducing suicidal behaviors.

Regarding the risk factors of suicide attempts, participants who had depression were three times more likely to have suicidal attempts as compared to non-depressed ones. The finding was consistent with other studies done in Vietnamese, English, Thailand, and Nigeria [66, 70, 71]. This is due to decreased serotonin levels in the brains of depressed individuals are associated with an increased risk of suicide attempts [72]. Adolescent girls with depression often exhibit symptoms such as hopelessness, helplessness, and isolation core features of demoralization, which can lead to suicidal attempts [73]. Moreover, adolescent girls with depressive symptoms may have a loss of interest in activities, negative self-perception, a tendency to experience the world as hostile and demanding, and a future expectation of suffering and failure, eventually leading to suicide attempts [72]. Therefore, giving mental health services in the community for depressed individuals is a crucial means to reduce suicidal ideation.

The odds of having suicidal attempts were about 4.6 times more likely among adolescent girls who had experienced gender-based violence than among participants who had not experienced gender-based violence. This result was in line with studies done in the USA [59], Cambodia [60], Peru [49], Ghana [61], Brazil [74], Malaysia [36], and Benin [75]. The associations may stem from GBV during this period, which has significant mental health implications for adolescent girls. These consequences include depression, anxiety, post-traumatic stress disorder (PTSD), substance abuse, chronic inflammation, and low self-esteem that lead to suicide [36, 59]. Thus, working on reducing the consequences and interventions that address the issue of GBV against adolescent girls is important to reduce suicidal attempts.

The odds of having suicidal attempts among participants who had a family history of suicide were two times higher as compared with the referent groups. Studies from Brazil [76], Tanzania [77], and Ethiopia [78] supported this. This may be due to the families and genetic linkage of suicide, as evidence reported [70, 76]. Another possible justification could be that family mental health issues present risk factors in offspring that elevate suicidal behaviors [79]. Furthermore, family members who have attempted suicide and victims are at a significantly increased risk of suicidal behavior on the anniversary [80].

Another associated factor with suicidal attempts was having a social phobia, which is 1.8 times more likely to have suicidal attempts than those who did not. This is consistent with a previous study conducted in the USA [81]. The reason could be that social anxiety is often associated with shyness, frustration, behavioral inhibition, overanxious disorder, school refusal, feelings of hopelessness, isolation, stigma, and low self-esteem that increase suicidal attempts [82]. This often leads to a chronic, unremitting course, leads to substantial impairments in vocational, and social functioning, substance use disorders, depression, and mental disorders increase suicide risk [81, 82]. This finding suggests that expanding mental health services in the community and addressing SP among adolescent girls are very important to preventing suicidal attempts.

## Limitation of the study

This study explores suicide and help-seeking behaviors among adolescent girls in Ethiopia. However, it has limitations. Firstly, the cross-sectional study design makes it difficult to establish cause-effect relationships between the outcome and explanatory variables. Secondly, the study did not assess the factors that influence help-seeking behaviors among adolescent girls. Finally, the magnitude of the problem may be underestimated due to the sensitive nature of topics like sexual violence and suicide, which can lead to social desirability bias. To mitigate this bias, female interviewers were also used and trained to be trauma-informed when asking sensitive questions for data collectors.

## Conclusions and recommendations

The study indicated that one in four adolescent girls had experienced suicidal ideation, one in six had attempted suicide, and help-seeking behaviors for suicidality were low. Being experienced gender-based violence, having anxiety symptoms, being food insecure, and having premenstrual dysphoric disorder were statistically significant predictors of suicidal ideation. The current study also revealed that suicidal attempts were higher among adolescent girls having depressive symptoms, having experienced gender-based violence, having a family history of suicide, and having social phobia. Since suicide needs urgent action, all concerned stakeholders should give better intervention. We recommend the Ministry of Health expand and implement mental health services in the community by designing universal, multi-level, collaborative, targeted intervention efforts especially by giving training and supporting front line primary care health staffs towards the assessment, diagnosis and management of common mental disorders, suicide prevention and encouraging help-seeking behaviors are crucial.

## Data Availability

The raw data used in this study are available upon request from the corresponding author.

## References

[1] Duke T, et al. World health organization and knowledge translation in maternal, newborn, child and adolescent health and nutrition. Arch Dis Child. 2022;107(7):644–9. 34969670 10.1136/archdischild-2021-323102 PMC7613575

[2] WHO. Preventing suicide: A global imperative. https://www.who.int/teams/mental-health-and-substance-use/overview; https://www.who.int/publications/i/item/9789241564779. Accessed on 17 Aug 2014.

[3] Bureau PR. 2008 WORLD POPULATION inform empower advance Population Reference Bureau Data Sheet. 2008. https://www.prb.org/wp-content/uploads/2008/08/2008-WPDS_Eng.pdf

[4] WHO. Suicide worldwide in 2019. Suicide worldwide in 2019. https://www.who.int/publications/i/item/9789240026643.

[5] PMA2020/ and, Ethiopia. Adolescents and young women health brief. 2020. https://www.pmadata.org/sites/default/files/data_product_results/PMA2020-Ethiopia-R4-Adol-Brief.pdf.

[6] Heilbron N, Prinstein MJ. Adolescent peer victimization, peer status, suicidal ideation, and nonsuicidal self-injury: examining concurrent and longitudinal associations. Merrill-Palmer quarterly. Wayne State University Press; 2010. 56: p. 388. 3. 10.1353/mpq.0.0049 PMC6103532 30147215

[7] Begum A, et al. Prevalence of suicide ideation among adolescents and young adults in rural Bangladesh. Int J Ment Health. 2017;46(3):177–87.

[8] Association AP. Diagnostic and statistical manual of mental disorders. Text revision; 2000.

[9] Jans T, Taneli Y, Warnke A. Suicide and self-harming behaviour. Proceedings of the Geneva, International Association for Child and Adolescent Psychiatry and Allied Professions; 2012.

[10] Nock MK, et al. Prevalence, correlates, and treatment of lifetime suicidal behavior among adolescents: results from the National comorbidity survey replication adolescent supplement. JAMA Psychiatr. 2013;70(3):300–10. 10.1001/2013.jamapsychiatry.55 PMC3886236 23303463

[11] Patton GC, et al. Global patterns of mortality in young people: a systematic analysis of population health data. Lancet. 2009;374(9693):881–92. 19748397 10.1016/S0140-6736(09)60741-8

[12] WHO. Suicide worldwide in 2019. 2021 [cited 2021 21 June]; Available from: https://www.who.int/publications/i/item/9789240026643

[13] McKinnon B, et al. Adolescent suicidal behaviours in 32 low-and middle-income countries. Bull World Health Organ. 2016;94(5):340. 27147764 10.2471/BLT.15.163295 PMC4850530

[14] Dixon PG, et al. Association of weekly suicide rates with temperature anomalies in two different climate types. Int J Environ Res Public Health. 2014;11(11):11627–44. 25402561 10.3390/ijerph111111627 PMC4245634

[15] Zygo M, et al. Prevalence and selected risk factors of suicidal ideation, suicidal tendencies and suicide attempts in young people aged 13–19 years. Ann Agric Environ Med. 2019;26(2):329–36. 31232067 10.26444/aaem/93817

[16] WHO. Suicide. 2024. https://www.who.int/news-room/fact-sheets/detail/suicide. Accessed on 29 August 2024.

[17] Kann L. Youth risk behavior surveillance—United States, 2017. MMWR. Surveillance Summaries; 2018. p. 67. 10.15585/mmwr.ss6708a1 PMC6002027 29902162

[18] Zoroglu SS, et al. Suicide attempt and self-mutilation among Turkish high school students in relation with abuse, neglect and dissociation. J Neuropsychiatry Clin Neurosci. 2003;57(1):119–26. 10.1046/j.1440-1819.2003.01088.x 12519464

[19] Auger N, et al. Suicide attempts in children aged 10–14 years during the first year of the COVID-19 pandemic. J Adolesc Health. 2023;72(6):899–905. 36870902 10.1016/j.jadohealth.2023.01.019 PMC9980433

[20] Glenn CR, Franklin JC, Nock MK. Evidence-based psychosocial treatments for self-injurious thoughts and behaviors in youth. J Clin Child Adolesc Psychol. 2015;44(1):1–29. 25256034 10.1080/15374416.2014.945211 PMC4557625

[21] Hom MA, Stanley IH, Joiner TE. Evaluating factors and interventions that influence help-seeking and mental health service utilization among suicidal individuals: a review of the literature. Clin Psychol Rev. 2015;40:28–39. 26048165 10.1016/j.cpr.2015.05.006

[22] Lucas W. World Mental Health Day: 10 October 2023.

[23] Pelkonen M, Marttunen M. Child and adolescent suicide: epidemiology, risk factors, and approaches to prevention. Pediatr Drugs. 2003;5:243–65. 10.2165/00128072-200305040-00004 12662120

[24] Pan C-H. Suicidal ideation, psychopathology, and help-seeking in 15 to 19-year-old adolescents in Taiwan: a population-based study 2015-2019. J Affect Disord. 2021;282:846–51. 33601727 10.1016/j.jad.2020.12.139

[25] Luoma JB, Martin CE, Pearson JL. Contact with mental health and primary care providers before suicide: a review of the evidence. Am J Psychiatry. 2002;159(6):909–16. 12042175 10.1176/appi.ajp.159.6.909 PMC5072576

[26] Michelmore L, Hindley P. Help-seeking for suicidal thoughts and self‐harm in young people: a systematic review. Suicide and Life-Threatening Behavior. 2012;42(5):507–24. 22889130 10.1111/j.1943-278X.2012.00108.x

[27] Rasmussen ML, Hjelmeland H, Dieserud G. Barriers toward help-seeking among young men prior to suicide. Death Stud. 2018;42(2):96–103. 28489969 10.1080/07481187.2017.1328468

[28] Miranda-Mendizabal A, et al. Gender differences in suicidal behavior in adolescents and young adults: systematic review and meta-analysis of longitudinal studies. Int J Public Health. 2019;64:265–83. 30635683 10.1007/s00038-018-1196-1 PMC6439147

[29] Lee E. Experiences of bereaved families by suicide in South Korea: a phenomenological study. Int J Environ Res Public Health. 2022;19(5):2969. 35270661 10.3390/ijerph19052969 PMC8910318

[30] Takahashi Y, et al. The National strategies for suicide prevention by the united Nation/World health organization and the present situation of suicide in the East Asia. Seishin Shinkeigaku Zasshi = Psychiatria Et Neurologia Japonica. 2014;116(8):690–6. 25244733

[31] Peterson C. *Economic cost of injury—United States, 2019.* MMWR. Morbidity and Mortality Weekly Report, 2021. 70. 10.15585/mmwr.mm7048a1 PMC8641568 34855726

[32] Organization WH. Suicide. 2024 [cited 2024 29 Augest]; Available from: https://www.who.int/news-room/fact-sheets/detail/suicide

[33] Holt MK, et al. Bullying and suicidal ideation and behaviors: A meta-analysis. Pediatrics. 2015;135(2):e496–509. 25560447 10.1542/peds.2014-1864 PMC4702491

[34] Miller AB, et al. The relation between child maltreatment and adolescent suicidal behavior: a systematic review and critical examination of the literature. Clin Child Fam Psychol Rev. 2013;16:146–72. 23568617 10.1007/s10567-013-0131-5 PMC3724419

[35] Joiner TE, et al. Childhood physical and sexual abuse and lifetime number of suicide attempts: a persistent and theoretically important relationship. Behav Res Ther. 2007;45(3):539–47. 16765909 10.1016/j.brat.2006.04.007

[36] Chan YY, et al. Prevalence and risk factors associated with suicidal ideation among adolescents in Malaysia. Int J Adolesc Med Health. 2018;30(3):20160053. 10.1515/ijamh-2016-0053 27508957

[37] Valdez-Santiago R, et al. Attempted suicide among adolescents in Mexico: prevalence and associated factors at the National level. Inj Prev. 2018;24(4):256–61. 28751532 10.1136/injuryprev-2016-042197

[38] Kessler RC, Üstün TB. The world mental health (WMH) survey initiative version of the world health organization (WHO) composite international diagnostic interview (CIDI). Int J Methods Psychiatr Res. 2004;13(2):93–121. 15297906 10.1002/mpr.168 PMC6878592

[39] Wilson CJ, et al. Measuring help-seeking intentions: properties of the general help seeking questionnaire. Can J Counselling. 2005;39(1):15–28.

[40] Rickwood D, et al. Young people’s help-seeking for mental health problems. Aust E-J Adv Ment Health. 2005;4(3):218–51.

[41] Johnson JG, et al. The patient health questionnaire for adolescents: validation of an instrument for the assessment of mental disorders among adolescent primary care patients. J Adolesc Health. 2002;30(3):196–204. 11869927 10.1016/s1054-139x(01)00333-0

[42] Manzar MD, et al. Psychometric properties of the generalized anxiety disorder-7 scale in Ethiopian university students. Bull Menninger Clin. 2021;85(4):405–27. 34851681 10.1521/bumc.2021.85.4.405

[43] Chukwujekwu D, Olose E. Validation of the social phobia inventory (Spin) in Nigeria. J Psychiatry Psychiatr Disord. 2018;2(2):49–54.

[44] Hussein FM, Ahmed AY, Muhammed OS. Household food insecurity access scale and dietary diversity score as a proxy indicator of nutritional status among people living with HIV/AIDS, Bahir Dar, Ethiopia, 2017. PLoS One. 2018;13(6):e0199511. 29953457 10.1371/journal.pone.0199511 PMC6023122

[45] Shayo FK, Lawala PS. Does food insecurity link to suicidal behaviors among in-school adolescents? Findings from the low-income country of sub-Saharan Africa. BMC Psychiatry. 2019;19:1–8. 31340781 10.1186/s12888-019-2212-6 PMC6657165

[46] Black DW, Grant JE. DSM-5^®^ guidebook: the essential companion to the diagnostic and statistical manual of mental disorders. American Psychiatric Pub; 2014.

[47] Gryczynski J, et al. Validation and performance of the A lcohol, S Moking and S Ubstance I Nvolvement S creening T est (ASSIST) among adolescent primary care patients. Addiction. 2015;110(2):240–7. 25311148 10.1111/add.12767 PMC4301997

[48] Dalgard OS, et al. Negative life events, social support and gender difference in depression: a multinational community survey with data from the ODIN study. Soc Psychiatry Psychiatr Epidemiol. 2006;41:444–51. 16572275 10.1007/s00127-006-0051-5

[49] Sharma B, et al. Factors associated with suicidal ideation and suicide attempt among school-going urban adolescents in Peru. Int J Environ Res Public Health. 2015;12(11):14842–56. 26610536 10.3390/ijerph121114842 PMC4661683

[50] Quarshie ENB, Onyeaka HK, Oppong Asante K. Suicidal behaviours among adolescents in Liberia. BMC Psychiatry. 2020;20(1):572. 33256674 10.1186/s12888-020-02985-3 PMC7706245

[51] Kapusta ND, et al. Availability of mental health service providers and suicide rates in Austria: a nationwide study. Psychiatr Serv. 2010;61(12):1198–203. 21123403 10.1176/ps.2010.61.12.1198

[52] Jordans M, et al. Suicidal ideation and behaviour among community and health care seeking populations in five low-and middle-income countries: a cross-sectional study. Epidemiol Psychiatr Sci. 2018;27(4):393–402. 28202089 10.1017/S2045796017000038 PMC5559346

[53] Uddin R, et al. Suicidal ideation, suicide planning, and suicide attempts among adolescents in 59 low-income and middle-income countries: a population-based study. Lancet Child Adolesc Health. 2019;3(4):223–33. 30878117 10.1016/S2352-4642(18)30403-6

[54] Peltzer K, Pengpid S. Early substance use initiation and suicide ideation and attempts among school-aged adolescents in four Pacific Island countries in Oceania. Int J Environ Res Public Health. 2015;12(10):12291–303. 26437423 10.3390/ijerph121012291 PMC4626969

[55] Kann L, et al. Youth risk behavior surveillance - United States, 2017. MMWR Surveill Summ. 2018;67(8):1–114. 29902162 10.15585/mmwr.ss6708a1 PMC6002027

[56] Zoroglu SS, et al. Suicide attempt and self-mutilation among Turkish high school students in relation with abuse, neglect and dissociation. Psychiatry Clin Neurosci. 2003;57(1):119–26. 12519464 10.1046/j.1440-1819.2003.01088.x

[57] Pengpid S, Peltzer K. Prevalence and correlates of past 12-month suicide attempt among in-school adolescents in Guatemala. Psychol Res Behav Manag. 2019;12. 10.2147/PRBM.S212648. 31372072 10.2147/PRBM.S212648 PMC6628607

[58] Badarch J, et al. Suicide attempts among school-attending adolescents in Mongolia: associated factors and gender differences. Int J Environ Res Public Health. 2022;19(5):2991. 35270685 10.3390/ijerph19052991 PMC8910274

[59] Salzinger S, et al. Adolescent suicidal behavior: associations with preadolescent physical abuse and selected risk and protective factors. J Am Acad Child Adolesc Psychiatry. 2007;46(7):859–66. 17581450 10.1097/chi.0b013e318054e702

[60] Caballero-Domínguez CC, Campo-Arias A. Prevalence and factors associated with suicide ideation in Colombian Caribbean adolescent students. OMEGA. 2022;85(4):837–49. 32921257 10.1177/0030222820959929

[61] Asante KO, et al. The prevalence and correlates of suicidal behaviours (ideation, plan and attempt) among adolescents in senior high schools in Ghana. SSM-population Health. 2017;3:427–34. 29349236 10.1016/j.ssmph.2017.05.005 PMC5769048

[62] Devries K et al. Violence against women is strongly associated with suicide attempts: evidence from the WHO multi-country study on women’s health and domestic violence against women. Soc Sci Med. 2011. 73 (1) pp. 79–86 10.1016/j.socscimed.2011.05.006 21676510

[63] Stark L, Ager A. A systematic review of prevalence studies of gender-based violence in complex emergencies. Trauma Violence Abuse. 2011;12(3):127–34. 21511685 10.1177/1524838011404252

[64] Beyene AS, et al. Gender-based violence among female youths in educational institutions of sub-Saharan Africa: a systematic review and meta-analysis. Syst Rev. 2019;8:1–14. 30803436 10.1186/s13643-019-0969-9 PMC6388495

[65] Yen C-F, et al. The associations between suicidal ideation and attempt and anxiety symptoms and the demographic, psychological, and social moderators in Taiwanese adolescents. Arch Suicide Res. 2014;18(1):104–16. 24354459 10.1080/13811118.2013.824826

[66] Nguyen DT, Wright EP. Low self-esteem and its association with anxiety, depression, and suicidal ideation in Vietnamese secondary school students: a cross-sectional study. Front Psychiatry. 2019;10:438641. 10.3389/fpsyt.2019.00698 PMC6777005 31611825

[67] Pilver CE, Libby DJ, Hoff RA. Premenstrual dysphoric disorder as a correlate of suicidal ideation, plans, and attempts among a nationally representative sample. Soc Psychiatry Psychiatr Epidemiol. 2013;48:437–46. 22752111 10.1007/s00127-012-0548-z PMC3774023

[68] Hong JP, et al. Prevalence, correlates, comorbidities, and suicidal tendencies of premenstrual dysphoric disorder in a nationwide sample of Korean women. Soc Psychiatry Psychiatr Epidemiol. 2012;47:1937–45. 22538387 10.1007/s00127-012-0509-6

[69] Wittchen H-U, et al. Prevalence, incidence and stability of premenstrual dysphoric disorder in the community. Psychol Med. 2002;32(1):119–32. 11883723 10.1017/s0033291701004925

[70] Glowinski AL, et al. Suicide attempts in an adolescent female twin sample. J Am Acad Child Adolesc Psychiatry. 2001;40(11):1300–7. 11699804 10.1097/00004583-200111000-00010 PMC1474069

[71] Adewuya AO, Oladipo EO. Prevalence and associated factors for suicidal behaviours (ideation, planning, and attempt) among high school adolescents in Lagos, Nigeria. Volume 29. European child & adolescent psychiatry; 2020. pp. 1503–12. 11. 10.1007/s00787-019-01462-x 31858265

[72] O’Connor RC, Rasmussen S, Hawton K. Predicting depression, anxiety and self-harm in adolescents: the role of perfectionism and acute life stress. Behav Res Ther. 2010;48(1):52–9. 19818954 10.1016/j.brat.2009.09.008

[73] Carter R, et al. Measures matter: the relative contribution of anxiety and depression to suicidal ideation in clinically referred anxious youth using brief versus full length questionnaires. Depress Anxiety. 2008;25(8):E27–35. 18729149 10.1002/da.20468

[74] Sousa CMdS, et al. Suicidal ideation and associated factors among high school adolescents. Rev Saude Publica. 2020;54:33. 32236384 10.11606/s1518-8787.2020054001637 PMC7100950

[75] Randall JR, et al. Suicidal behaviour and related risk factors among school-aged youth in the Republic of Benin. PLoS One. 2014;9(2):e88233. 24505443 10.1371/journal.pone.0088233 PMC3914941

[76] Sousa CMdS, et al. Ideação suicida e fatores associados entre escolares adolescentes. Volume 54. Revista de Saúde Pública; 2020. p. 33.

[77] Dunlavy AC, Aquah EO, Wilson ML. Suicidal ideation among school-attending adolescents in Dar Es Salaam, Tanzania. Tanzan J Health Res, 2015. 17(1). page 12-16

[78] Kassa MA, et al. Suicidal ideation and attempts among high school students of war-affected area at Woldia town, Northeast, Ethiopia, 2022. BMC Psychiatry. 2023;23(1):384. 37259028 10.1186/s12888-023-04889-4 PMC10234009

[79] Simbar M, et al. Suicide risk factors in adolescents worldwide: a narrative review. J Rafsanjan Univ Med Sci. 2018;16(12):1153–68.

[80] Jang S-I, et al. The effect of suicide attempts on suicide ideation by family members in fast developed country. Korea. Compr Psychiatry. 2016;66:132–8. 26995246 10.1016/j.comppsych.2016.01.008

[81] Gallagher M, et al. Social anxiety symptoms and suicidal ideation in a clinical sample of early adolescents: examining loneliness and social support as longitudinal mediators. J Abnorm Child Psychol. 2014;42:871–83. 24390470 10.1007/s10802-013-9844-7 PMC5496444

[82] Lynch H, McDonagh C, Hennessy E. Social anxiety and depression stigma among adolescents. J Affect Disord. 2021;281:744–50. 33257039 10.1016/j.jad.2020.11.073

